# Comprehensive improvements in the emergency laboratory test process based on information technology

**DOI:** 10.1186/s12911-023-02387-x

**Published:** 2023-12-19

**Authors:** Liang Zhang, Zhen Hua Liu, Yin Jiang Lv, Shui Fu, Zhang Mei Luo, Mei Li Guo

**Affiliations:** 1 Department of Clinical Laboratory, The Second Affiliated Hospital of Zhejiang University School of Medicine, Linping Campus, 311100 Hangzhou, Zhejiang Province People’s Republic of China; 2 Department of General Surgery, The Second Affiliated Hospital of Zhejiang University School of Medicine, Linping Campus, 311100 Hangzhou, Zhejiang Province People’s Republic of China; 3 Department of Clinical Laboratory, The People’s Hospital of Cangnan Zhejiang, No. 2288 Yucang Road, Cangnan County, 325800 Wenzhou, Zhejiang Province People’s Republic of China

**Keywords:** Information technology, Emergency laboratory test, Process optimisation, Satisfaction, Turn-around time

## Abstract

**Objective:**

To explore the application effects of information technology (IT) on emergency laboratory testing procedures.

**Methods:**

In this study, IT-based optimisation of the emergency laboratory testing process was implemented between October and December 2021. Thus, the emergency laboratory test reports from January to September 2021 were placed into the pre-optimised group, while those from January to September 2022 were categorised into the post-optimised group. Besides, the emergency laboratory test report time, emergency laboratory test report time limit coincidence rate, error rate, and employee and patient satisfaction levels in individual months and across the whole period were described. Moreover, changes in the above indicators before and after the implementation of IT-based optimisation were explored and the application effects of IT-based optimisation were also evaluated.

**Results:**

The emergency laboratory test report times after the implementation of IT-based optimisation were shorter than those before IT-based optimisation (*P* < 0.05). The total number of laboratory test items before and after information optimization amounted to 222,139 and 259,651, respectively. Also, IT-based optimisation led to an increase in the emergency laboratory test report time limit coincidence rate from 98.77% to 99.03% (*P* < 0.05), while the emergency laboratory test report error rate fell from 0.77‱ to 0.15‱ (*P* < 0.05). Additionally, IT-based optimisation resulted in increases in both employee satisfaction, from 80.65% to 93.55% (N = 31, *P* > 0.05), and patient satisfaction, from 93.06% to 98.44% (*P* < 0.05).

**Conclusion:**

The automation and IT-based optimisation of the emergency laboratory testing process significantly reduces the emergency laboratory test report time and error rate. Additionally, IT-driven optimization enhances the alignment of emergency laboratory test report deadlines and enhances the overall quality and safety of emergency laboratory testing.

## Introduction

With the progress of medical reforms in China, there is now an increasing demand for high-quality healthcare services. Stricter requirements regarding the quality and efficiency of laboratory tests have also been proposed in clinical practice. On that basis, to further improve the quality and speed of laboratory test reports, clinicians are exploring methods to satisfy the demands 

of clinical applications and patients for the accurate and timely processing of laboratory tests. In China, most patients with acute onset, severe symptoms, or complex conditions are initially admitted to the emergency department, which increases the aggregation of patients with acute and critical conditions [1]. In contrast to conventional patients, patients in the emergency department often simultaneously require emergency rescue and disease diagnosis. Therefore, the emergency department must provide the ideal allocation of limited resources in the shortest possible time. Additionally, there are more stringent requirements for emergency laboratory test results, since delayed or inaccurate test results may affect the clinical diagnosis and lead to delayed or even inappropriate treatment [2, 3]. Emergency laboratory tests are a vital component of emergency medical treatment. A comprehensive array of emergency laboratory test items and accurate and timely test reports provide adequate support for emergency patients to receive effective care within the optimal rescue window [4, 5]. Moreover, the rapid evaluation of patients can be achieved within the emergency department in case of sudden onset of disease, trauma, or public health emergencies. Meanwhile, the emergency department should cooperate with various hospital departments to make clinical decisions swiftly, thereby saving the lives of patients and avoiding further aggravation of disease. As the front line of emergency diagnosis and treatment, emergency laboratory tests play a crucial role in the treatment of patients with acute, severe, and critical conditions. Moreover, emergency laboratory test results exert a significant impact on making timely and accurate clinical decisions [6]. Therefore, reducing emergency laboratory test report times is essential for enhancing emergency treatment capability.

According to the *Accreditation Criteria for the Quality and Competence of Medical Laboratories* (ISO15189) [7], turnaround time (TAT) is defined as “the duration between two designated points in the process before, during, and after laboratory tests”. TAT is employed to reflect the working efficiency of laboratories and is the preferred indicator for evaluating laboratory service quality. The laboratory test report time refers to the laboratory TAT, namely the duration (in min) from the time when the laboratory receives the specimen to the time when the report is sent. As a result, TAT has become an important controllable quality indicator for laboratory testing. There are specific regulations and requirements regarding this indicator in the *Laboratory Management Measures and Detailed Rules and Regulations for the Implementation of Evaluation Standards for Tertiary General Hospitals* (2011). Additionally, TAT was included as one of the 15 quality indicators issued by the National Centre for Clinical Laboratories in 2015 and it has been verified as an important quality indicator in laboratory tests. As specified in *Accreditation Criteria for the Quality and Competence of Medical Laboratories* (ISO 15,189) [8], laboratory quality management includes the management of test result authenticity and reliability and also covers the management of various factors that may affect test results. The laboratory test report time is a key factor that affects the diagnosis and treatment of patients. In numerous clinical laboratories worldwide, the laboratory test report time is regarded as an important observation indicator for continuous quality improvement, while limitations in the emergency laboratory test report time have been highlighted [9–11].

The application range of laboratory information technology has become an important symbol of scientific laboratory quality management. By applying information management technology to analyse and monitor the time nodes of key links in laboratory tests, the main test line, namely the “laboratory test report”, can be more effectively managed. Although IT has been applied to laboratory management and achieved favourable results [12–14], transformation and optimisation based on this technology are performed differently according to the procedures of individual hospitals. Hence, it is necessary to incorporate IT into the overall emergency laboratory test process and constantly conduct summarisations and optimisations in clinical practice, thereby maximising automation and early warning during each step. Based on this, the working efficiency can be improved and the laboratory test report time can be reduced, which is consistent with the core values of the hospital: “Focus on the perceptions of patients and employees”. This scientific study lays a solid foundation for appropriately applying emergency laboratory tests in clinical practice, enhancing the quality of medical services, ensuring patient safety, and improving patient satisfaction.

## Data and methods

### General data

As a contemporary tertiary second-class general hospital, our institution seamlessly integrates medical treatment, scientific research, teaching, rehabilitation, prevention, and healthcare services. We cater to approximately 1.8 million outpatients and admit around 50,000 inpatients annually. The hospital encompasses a sprawling site area of 108 mu, with a combined utilization area totaling 140,000 square meters. Our facilities include 32 open wards, housing a total of 1,000 beds, and a dedicated workforce of over 1,500 employees. In our department, the IT-based optimisation of the emergency laboratory test process was implemented between October and December 2021. The emergency laboratory test reports from January to September 2021 were assigned to the pre-optimisation group, while those from January to September 2022 were categorised into the post-optimisation group. The emergency laboratory test report time limit for routine blood test + CRP, routine urine test, routine faecal test, blood group test, urine pregnancy test, and blood gas analysis was ≤ 30 min, while that of biochemical, immune, and other tests was ≤ 1 h.

### Methods

#### Materials

A Laboratory Information Management System (LIS) was purchased from Hangzhou Lingyun Technology Co. Any problems encountered in emergency laboratory tests were discussed within the department and then reported to software engineers. Subsequently, IT engineers conducted repairs and optimisations to solve these issues.

#### Circumstances and problems in the laboratory test process before optimisation

**Fig. 1:**
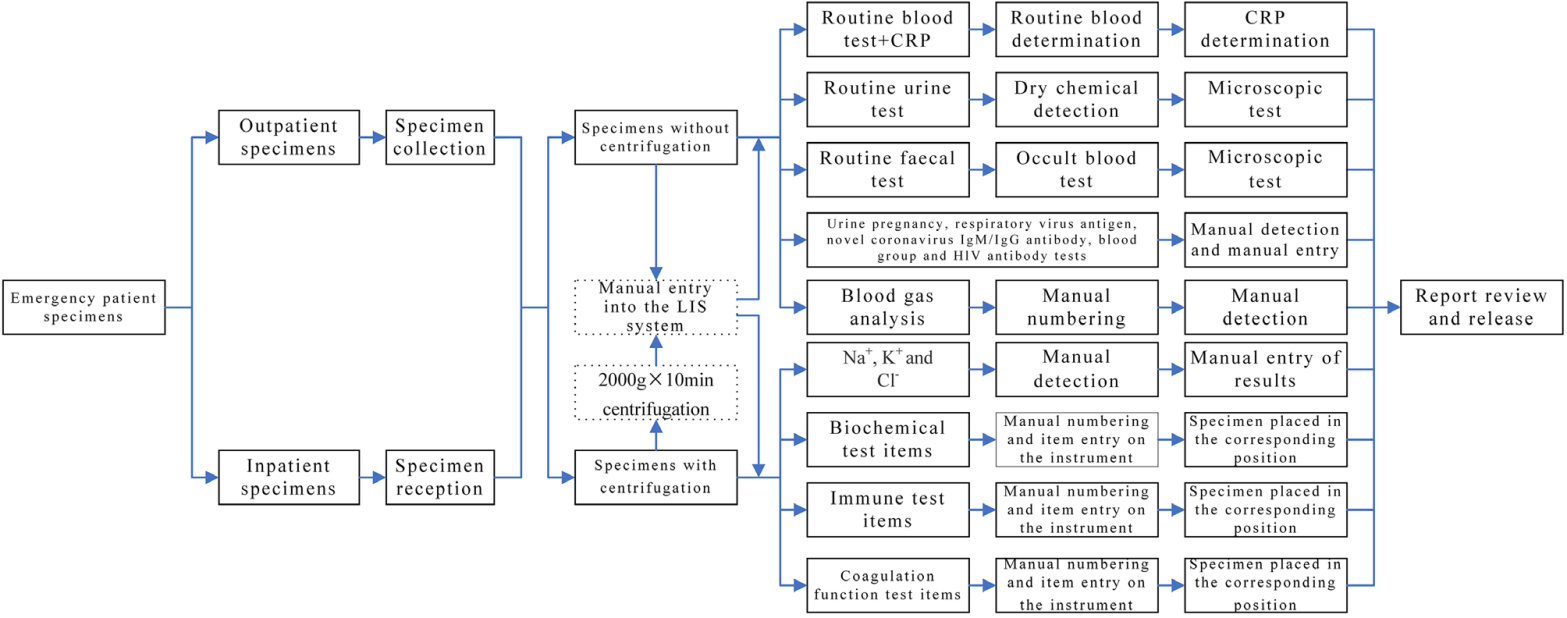
Emergency laboratory test workflow before IT-based optimisation

Specimens obtained from patients in outpatient or emergency departments labeled as “emergency” and collected by clinicians in clinical departments for laboratory tests are referred to as non-hospitalised specimens. In contrast, specimens collected from inpatients with “emergency” labels in clinical departments for laboratory tests are designated as hospitalised specimens. The emergency laboratory test specimens of outpatients were manually entered into the corresponding category in the LIS system, with a category specified for each item. However, for inpatients, the emergency laboratory test specimens were first input into the receiving system and then manually entered into the corresponding category in the LIS system. Subsequently, an automated or manual test was conducted. The results were then submitted and reports reviewed as necessary, while all problems were resolved according to the operating procedures. The specific laboratory test items are listed in Table 1 and the detailed workflow is presented in Fig. 1.

**Table 1:** Emergency laboratory test items and corresponding detection instruments and reagents

Item Name	Sub-item	Instrument and Reagent	
		Before optimisation	After optimisation
Routine blood test + CRP (1 or 2 items)	Routine blood test	Sysmex XN-B4 (Sysmex, Japan)	Automatic Blood Cell Analyzer BC-7500CRP (Shenzhen Mindray Bio-Medical Electronics Co., Ltd.)
	CRP	Immune Analyzer Jet-iStar3000 (Joinstar Biomedical Technology Co., Ltd.)	
Routine urine test	Routine urine test	GEB-600 Urine Chemistry Analyzer (Guangzhou Huadu Gaoerbao Biotechnology Co., Ltd.)	Sysmex Urine Analysis Modular Assembly Line (Sysmex, Japan)
Routine faecal test	Routine faecal test	Semi-quantitative pyramidon method (Zhuhai Baso Diagnostics Inc.)	Semi-quantitative pyramidon method (Zhuhai Baso Diagnostics Inc.)
Respiratory influenza virus antigen (1 to 5 items)	Influenza A virus, influenza B virus, respiratory syncytial virus, adenovirus, and mycoplasma pneumoniae	Hangzhou Genesis Biodetection & Biocontrol Co., Ltd.	Hangzhou Genesis Biodetection & Biocontrol Co., Ltd.
Novel coronavirus antibody	Novel coronavirus IgM/IgG antibody	Nanjing Vazyme Biotech Co., Ltd.	Nanjing Vazyme Biotech Co., Ltd.
Urine pregnancy test	Urine pregnancy test	Blue Cross Bio-Medical (Beijing) Co., Ltd.	Blue Cross Bio-Medical (Beijing) Co., Ltd.
ABO + Rh blood group identification	ABO blood group identification	Shanghai Hemo Pharmaceutical & Biomedical Co., Ltd	Hamilton Automatic Blood Grouping Instrument (Hamilton, Switzerland)
	RhD blood group identification	Jiangsu Libo Medicine Biotechnology Co., Ltd.	
Blood gas analysis	Blood gas analysis	ABL90 FLEX Blood Gas Analyzer (Radiometer, Denmark)	ABL90 FLEX Blood Gas Analyzer (Radiometer, Denmark)
Electrolyte test	Electrolytes (Na^+^, Cl^−^, and K^+^)	Electrolyte Analyzer XD 690I (Shanghai Xunda Medical Instrument Co., Ltd.)	Automatic Biochemical Analyzer DxC 700 AU (Beckman Coulter, USA)
Biochemical test	Liver function (ALT and AST), renal function (urea and creatinine), myocardial zymogram (AST, lactate dehydrogenase, creatine kinase, and creatine kinase isoenzyme), blood glucose, amylase, and cholinesterase	Accute TBX-40FR (Tosoh Bioscience Shanghai Co., Ltd.)	
Biochemical test (new)	Total bilirubin, direct bilirubin, indirect bilirubin, total protein, and albumin globulin	Not performed	
Immune test (1 or 2 items)	TnI and β-HCG	Automatic Immune Analyzer AIA-360 (Tosoh Bioscience Shanghai Co., Ltd.)	Anility i Automatic Chemiluminescence Immunoassay Analyzer (Abbot, USA)
Four-item pre-surgery test	HIV antigen and antibody, treponema pallidum antibody, hepatitis C antibody, hepatitis B virus surface antigen, hepatitis B virus surface antibody, hepatitis B virus e antigen, hepatitis B virus e antibody, and hepatitis B virus core antibody	Not performed	
BNP test	BNP	Not performed	
HIV antibody test	Human immunodeficiency virus (HIV1 + 2) antibody	Beijing Wantai Biological Pharmacy	Beijing Wantai Biological Pharmacy
Coagulation function (1 to 5 items)	Coagulation function (PT, APTT, FIB, TT, and D-dimer)	Sysmex CA-1500 (Sysmex, Japan)	Sysmex CS-2000i (Sysmex, Japan)

Note: (1) After IT-based optimisation, the electrolyte test, biochemical test, and (new) biochemical test were combined into one biochemical test. The immune test, four-item pre-surgery test, and BNP were combined into one immune test. (2) Before IT-based optimisation, there were 13 test items and 36 test sub-items. After optimisation, there were 12 test items and 50 sub-items

##### Existing problems

(1) The instruments were outdated since most of them were purchased during the relocation of our hospital in 2013. Since a majority of the instruments were not equipped with two-way communication, it was necessary to enter the test items and record the test results manually in some instruments. (2) The specimen’s name could only be entered by manual code scanning, which amplified the workload and increased the likelihood of missing entries or incorrect set entries. (3) There were no relevant prompts throughout the whole process and no relevant tracking function for adverse events such as sample omission or timeout. (4) The items with critical values were reported by manual telephone notification or manual registration, which was time-consuming and also led to data entry errors (Table 2).

**Table 2:** Issues with emergency laboratory tests before IT-based optimisation and corresponding solutions

Problems	Solutions
All specimens are manually entered into the corresponding category of the LIS system, which increases the workload and may lead to incorrect categorisation or missing entries.	The LIS system numbers for outpatient specimens are automatically generated during collection, while those for inpatient specimens are automatically generated on reception of the specimens. Therefore, the number of specimens is generated automatically, which reduces the workload of employees and prevents errors.
The electrolyte (Na^+^, Cl^−^, and K^+^) test results are entered manually.	After the instruments are updated, these test items are integrated into the biochemical test and two-way communication is realised.
The biochemical, immune, and coagulation function test specimens are numbered manually before detection on the instrument. Also, these specimens are manually placed in their corresponding positions, which increases the workload and may result inhuman error.	Upgraded instruments allow the implementation of IT methods that realise automated detection and two-way communication.
During the manual identification of blood groups, the manual entry of results increases the workload and may cause human error.	After the purchase of automated equipment, IT methods are employed to realise detection automation and two-way communication.
The test items are relatively scattered and it is necessary to collect several specimen tubes to facilitate detection.	The electrolyte (Na^+^, Cl^−^, and K^+^) test items are integrated into the biochemical test and the HIV antigen and antibody test items are added to the immune test to replace the human immunodeficiency virus (HIV1 + 2) antibody test item. One tube of specimens is collected for the biochemical test to reduce the number of blood collection tubes.
The items with critical values are reported by manual telephone notification and manual registration.	The information system is optimised to generate critical value system report notifications and achieve constant monitoring. If the recipient fails to accept the notification within the specified time, an alarm prompt is sent to the target computer. Manual notification by telephone is performed if the recipient fails to accept it within 5 min of the alarm prompt display.
There is no prompt function for the timeout of a specimen report.	This real-time laboratory specimen monitoring system displays the specimen report with a timeout on the screen. Specimen reports with timeouts are highlighted and signalled with an alarm.
The layout in the laboratory is inappropriate and the placement of computers and equipment is not conducive to operation.	Based on the layout of higher-level hospitals, all employees in the department formulate feasible schemes according to the actual situation in this department. The layout of the laboratory is redesigned scientifically to facilitate operations.
The ABO blood group identification is performed manually and there is no reverse grouping.	The Hamilton automatic blood group identification instrument increases the reverse grouping function and realises automatic detection. This reduces manual operation and ensures rapid and accurate blood group identification.
Invalid symbols such as “-”, “*”, and “0”are sent to the LIS system in case of uncertain or undetected test results. The laboratory test report can be reviewed but there is no relevant prompt.	The LIS system is optimised. If there is an invalid symbol in the laboratory test report, a prompt is displayed during the report review and the review is performed after revisions.

#### Identification of problems in clinical practice and relevant optimisations

**Fig. 2:**
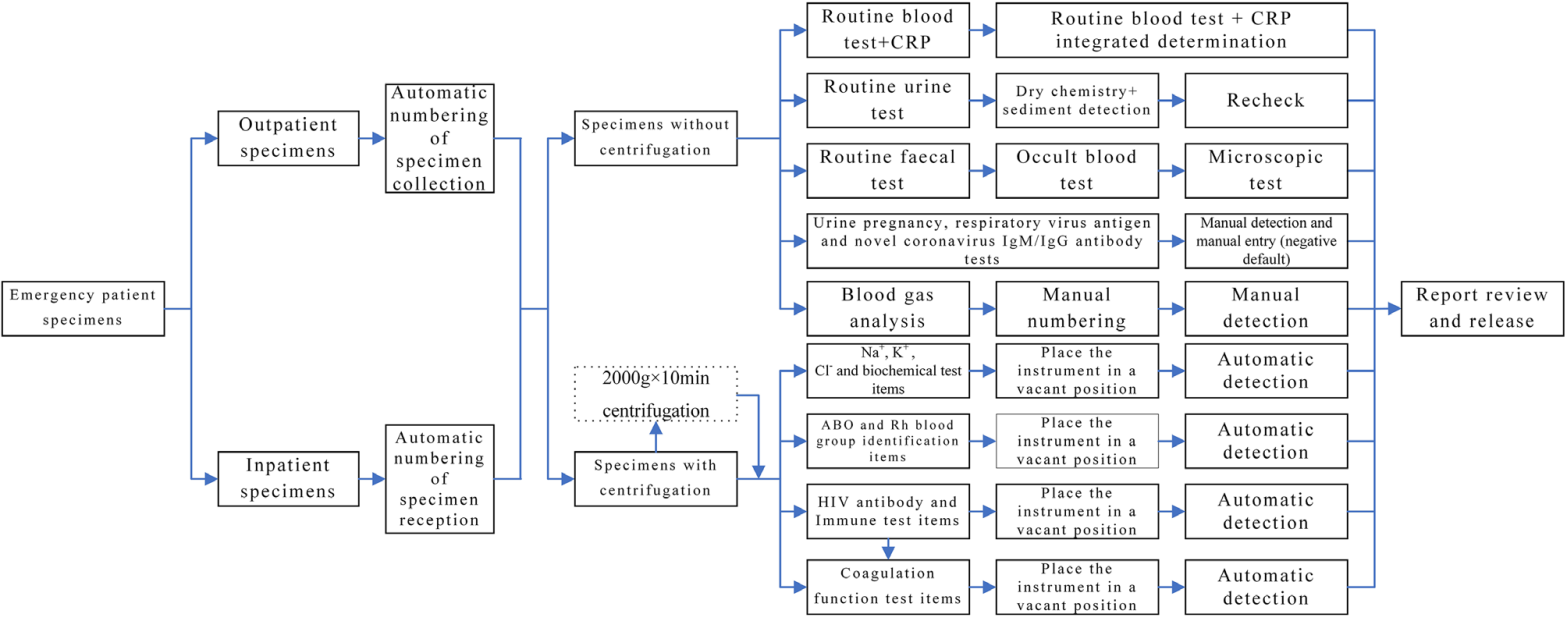
Emergency laboratory test workflow after IT-based optimisation

(1) Update equipment: The problems associated with instrument operation were dealt with. Some instruments were equipped with two-way communication with the aid of the manufacturer and the LIS system engineer. Besides, some new instruments were purchased to replace the existing ones, and all new instruments were equipped with two-way communication during installation. After automation and information integration of the equipment across the whole process was realised, manual numbering, manual entry of test items, and secondary entry of test results during instrument operation were eliminated. Meanwhile, various errors caused by manual operation were also resolved, thereby improving working efficiency and laboratory test quality; (2) Set up relevant prompt functions: According to the actual situation in the department, real-time specimen monitoring software was created to display upcoming timeout items (within 5 min) and timeout items (sound alarm) with the aid of a display screen and audio installed in a conspicuous position; (3) Automatic reports of the items with critical values and monitoring of the whole process: After the critical value report system was optimised, inspectors rechecked the test results in cases with prompts on items with critical values. After the test results were checked, they were manually entered into the LIS system and sent to the prescriber. If the recipient failed to accept the notification within the specified time, an alarm prompt was sent to the target computer. Manual notification by telephone was performed if the recipient failed to accept the notification within 5 min of the alarm prompt. These measures eliminated human error and timeouts in the manual reporting of items with critical values; (4) IT-based optimisation: The laboratory test items were optimised according to clinical practice. The blood collection tubes were integrated to reduce the volume of blood taken from patients and improve test efficiency. Additionally, problems encountered during daily operations were optimised individually, thereby contributing to user-friendly operation and close connection to the LIS system (Table 2). The related process is presented in Fig. 2.

### Observation indicators

In this study, relevant evaluation indicators were compared before and after optimisation. Laboratory test report time (outpatients) = Time when the test result is reported - Time when the specimen is collected; Laboratory test report time (inpatients) = Time when the test result is reported - Time when the specimen is accepted; Laboratory test report time limit coincidence rate = Number of laboratory test reports within the specified time limit/Total number of laboratory test reports × 100%; Laboratory test report error rate = Number of laboratory test reports with errors / Total number of laboratory test reports × 100%. Additionally, an anonymous satisfaction survey was performed on all employees involved in the emergency laboratory test process. *The Management System of Quality Monitoring Indicators of Medical Community* was developed in accordance with the standards outlined in the *Joint Commission International Accreditation Standards for Hospital* (6th Edition) [15]. Subsequently, 128 patients were randomly selected each month for the satisfaction survey. Satisfaction = Number of patients that are very satisfied or satisfied / Total number of patients in the satisfaction survey × 100%.

### Statistical analysis

Data processing was performed usingSPSS26.0 and the Kolmogorov-Smimov normality test was used to analyse the data distribution. The two-sample independent t-test was performed to make comparisons between both groups. Normally distributed measurement data were expressed using $\bar x$ ± s, while non-normally distributed measurement data were expressed using M (IQR). Moreover, the rank sum test was used for comparisons between groups that did not conform to normal distribution, and the chi-square test was performed on the comparison of rates. *P* < 0.05 indicated that there was a significant statistical difference.

## Results

### Changes in the emergency laboratory test report time before and after IT-based optimisation

After IT-based optimisation was implemented, the median emergency laboratory test report time in each month from January to September 2022 was shorter than in the corresponding months from January to September 2021 (i.e., January 2022 vs. January 2021), with statistically significant differences. After IT-based optimisation, the overall emergency laboratory test report time was also shorter than before optimisation, and the difference between the groups was significant (*P* < 0.05), as indicated by Table 3.

**Table 3:** Comparison of laboratory test report times before and after IT-based optimisation

Month	2021 (before optimisation)		2022 (after optimisation)		Z	P
	Number of items	Report time M (IQR) (min)	Number of items	Report time M (IQR) (min)		
January	23,936	28(22)	26,384	25(24)	-19.139	< 0.001
February	18,411	27(22)	17,598	24(24)	-11.516	< 0.001
March	26,436	29(23)	25,592	26(26)	-16.288	< 0.001
April	20,448	28(23)	28,012	25(24)	-20.460	< 0.001
May	21,366	29(23)	30,929	25(25)	-28.326	< 0.001
June	26,533	31(24)	32,894	27(25)	-33.300	< 0.001
July	34,820	30(23)	32,699	26(24)	-21.733	< 0.001
August	26,562	32(24)	33,126	27(26)	-39.652	< 0.001
September	23,627	30(25)	32,417	26(24)	-38.696	< 0.001
Total	222,139	30(24)	259,651	25(25)	-32.153	< 0.001

Note: There were13 test items before IT-based optimisation and 12 test items after optimisation

### Changes in the emergency laboratory test report time limit coincidence rate before and after IT-based optimisation

After IT-based optimisation, the total number of laboratory test items was 259,651 and the emergency laboratory test report time limit coincidence rate was 99.03%. In contrast, before optimisation, the total number of laboratory test items was 222,139 and the emergency laboratory test report time limit coincidence rate was 98.77%. The values of both indicators after IT-based optimisation were higher than before IT-based optimisation, and there was a significant difference in the emergency laboratory test report time limit coincidence rate (Table 4).

**Table 4:** Comparison of the emergency laboratory test report time limit coincidence rate before and after IT-based optimisation

Group	Total number of laboratory test reports (cases)	Total number of laboratory test reports satisfying time limit requirements (cases)	Coincidence rate (%)
Before optimisation	222,139	219,417	98.77
After optimisation	259,651	257,121	99.03
χ2	—	—	69.937
*P*	—	—	< 0.001

### Changes in the emergency laboratory test report error rate before and after IT-based optimisation

Before IT-based optimisation, there were 222,139 laboratory test items in total, with errors occurring in 17 laboratory test items, equating to 0.77‱. Specifically, there were five flawed test items caused by manual input errors, eight mistakes due to invalid symbol reports, two errors caused by poor specimen collection, one item with incorrect results caused by condensation, and one test item where CK-MB = 0. After IT-based optimisation, there were 259,651 laboratory test items in total, and errors only occurred in four laboratory test items, accounting for 0.15‱. Specifically, there was one faulty laboratory test item in routine faecal microscopic tests, one error concerning a higher D-dimer level caused by poor specimen collection, and two mistakes related to low RBC (Red Blood Cell, RBC) levels caused by condensation. After the implementation of IT-based optimisation, the emergency laboratory test report error rate decreased considerably, and there was a significant difference in this indicator between the two groups (Tables 5 and 6).

**Table 5:** Comparison of the emergency laboratory test report error rates before and after IT-based optimisation

Group	Total number of laboratory test reports (cases)	Total number of laboratory test reports with errors (cases)	Error rate (‱)
Before optimisation	222,139	17	0.77
After optimisation	259,651	4	0.15
χ2	—	—	8.907
*P*	—	—	0.002

**Table 6:** Causes of emergency laboratory test report errors

Error cause	Number before optimisation	Number after optimisation	Total
Manual entry error	5	1	6
“-” or “*” is displayed for the report results of quantitative items	8	0	8
Specimen collection before analysis	2	1	3
Specimen condensation	1	2	3
Laboratory test item result is 0	1	0	1
Total	17	4	21

### Comparison of patient and employee satisfaction before and after IT-based optimisation

**Fig. 3:**
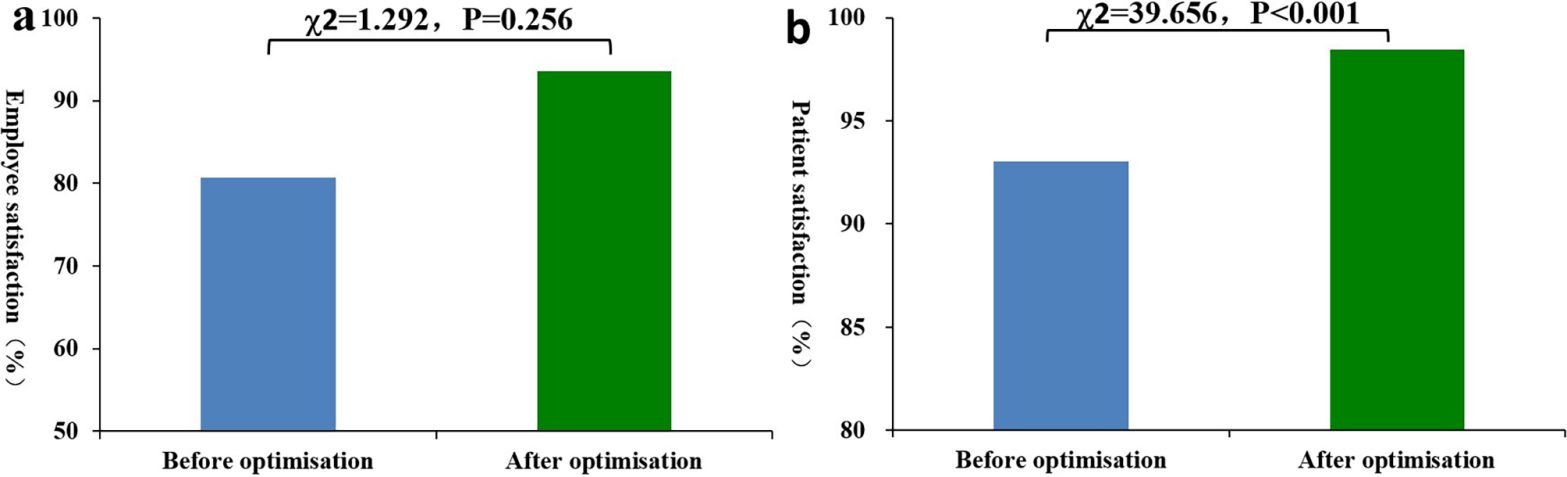
Satisfaction levels of employees and patients before and after IT-based optimisation

The satisfaction levels of employees involved in emergency laboratory tests (rated very satisfied or satisfied) increased from 80.65 to 93.55% after the introduction of IT-based optimisation, but the difference was not statistically significant (*P* = 0.256). However the number of very satisfied employees increased by 29.03% (from 54.84 to 83.87%) after IT-based optimization, and the difference was statistically significant (*P* = 0.025). After IT-based optimisation, patient satisfaction (very satisfied or satisfied) increased from 93.06 to 98.44%, exhibiting a statistically significant difference (*P* < 0.001). Additionally, after IT-based optimisation, the number of employees and patients who were very satisfied with the emergency laboratory tests increased significantly (employees: 17 occurring before and 26 after IT-based optimisation;(patients: 940 occurring before and 1044 after IT-based optimisation), and the difference was statistically significant (employees: *P* = 0.025, patients: *P* < 0.001). Besides, after IT-based optimisation, the number of patients who were satisfied, generally satisfied, and dissatisfied decreased substantially, and the differences were also statistically significant (*P* = 0.003, *P* < 0.001, *P* < 0.001). However, for these three satisfaction levels, there was no significant difference in the satisfaction of employees involved in emergency laboratory tests before and after IT-based optimisation (*P* = 0.096, *P* < 0.554, *P* < 0.554), see Tables 7 and 8, and Fig. 3.

**Table 7:** Comparison of the satisfaction of employees involved in emergency laboratory tests before and after IT-based optimisation

Group	Total number (cases)	Very satisfied (cases/%)	Satisfied (cases/%)	Generally satisfied (cases/%)	Dissatisfied (cases/%)	Satisfaction Rate
Before optimisation	31	17/54.84	8/25.81	4/12.90	2/6.45	80.65
After optimisation	31	26/83.87	3/9.68	1/3.23	1/3.23	93.55
χ2	—	5.01	2.763	0.35	0.35	1.292
*P*	—	0.025	0.096	0.554	0.554	0.256

**Table 8:** Comparison of patient satisfaction before and after IT-based optimisation

Group	Total number (cases)	Very satisfied (cases/%)	Satisfied (cases/%)	Generally satisfied (cases/%)	Dissatisfied (cases/%)	Satisfaction Rate
Before optimisation	1,152	940/81.60	132/11.46	60/5.21	20/1.74	93.06
After optimisation	1,152	1,044/90.63	90/7.81	16/1.39	2/0.17	98.44
χ2	-	39.252	8.793	26.343	13.263	39.656
*P*	-	< 0.001	0.003	< 0.001	< 0.001	< 0.001

## Discussion

To obtain precise and accurate medical test results, most laboratories highlight analytical techniques and test quality. However, since clinicians also pay attention to service quality and the timeliness of laboratory test reports, they have stricter requirements for emergency laboratory test reports [16, 17]. As specified in the *Accreditation Criteria for the Quality and Competence of Medical Laboratories* (ISO15189) [7], laboratory quality management encompasses management of the authenticity and reliability of test results and also covers the management of various factors that may affect these results. The TAT of specimens is a crucial factor that affects the quality of laboratory tests. Currently, TAT is regarded as an observation indicator for continuous quality improvement in numerous clinical laboratories around the world [9, 18]. With the rapid advancement and popularisation of IT, there have been tremendous improvements in the function of the hospital information system (HIS) [19] and clinical laboratory information system (LIS) [20] in hospitals at all levels. Besides, management of the conventional laboratory test process (before, during, and after analysis) has been continually improved. If the most advanced IT can be applied to medical laboratory testing, the quality and speed of these tests may be greatly improved. Using information management technology to analyse and monitor the time nodes of key stages during the inspection process enhances effectiveness and controllability. As a result, the work efficiency and inspection quality are improved.

As a county-level general hospital (tertiary second-class hospital), our institution placed significant emphasis on continuous quality improvement during the re-evaluation process for tertiary second-class hospitals in 2021. Additionally, in 2013, our hospital relocated to its current site, which included a new hospital area equipped with relevant facilities. It is worth noting that by this time, many of the instruments in the clinical laboratory had reached their scheduled retirement dates and required systematic replacement. Due to said circumstances, the PDCA method was adopted to analyse relevant problems first, with a focus on improving the quality of emergency laboratory test reports. Subsequently, the PDCA tool was employed to identify the foundational framework of emergency laboratory test reports, particularly focusing on the aspect of delayed information construction. Building upon this foundation, a multifaceted approach was adopted, with IT as the focal point, to optimise the emergency laboratory test process. This optimisation encompassed various comprehensive methods, including personnel management, 7 S management, equipment innovation, and alignment with hospital values, all aimed at enhancing the quality of emergency laboratory test reports. While this study indeed emphasises the optimisation of IT, it is crucial to note that the conclusion should not be solely based on the improvement of IT alone. The enhancement in the quality of emergency laboratory test reports primarily results from the comprehensive measures implemented through IT-based initiatives.

In this study, IT methods were employed to optimise each step-in the emergency laboratory test process (Table 2). Even with an increased number of specimens, the laboratory test report time was still reduced by 16.67% (from 30 min [median] to 25 min [median]) after IT-based optimisation. Additionally, the TAT was much shorter than the 68 min required to generate emergency biochemical test results, as reported by Fei et al. [21]. However, this discrepancy may be caused by differences in the test items. Specifically, we included all test items in this study, while only emergency biochemical test items were included in their study. The upper limit of the biochemical test report times in our laboratory was 60 min, while the TAT coincidence rate in our laboratory reached 98.77% before IT-based optimisation. Additionally, the emergency laboratory test report time in this study was shorter than that in the study of Fei et al. This may be because precise management and IT were less advanced when they conducted their relevant studies. Moreover, under the premise that the number of specimens increased and the number of employees remained the same, the emergency laboratory test report time limit coincidence rate significantly improved. This finding suggests that IT played an important role in cutting the emergency laboratory test report time. Additionally, the emergency laboratory test report time limit coincidence rate after IT-based optimisation in this study was also slightly higher than the94.8% reported by Zhang et al. [22]. This may be because they paid attention to analysing the TAT coincidence rate and exploring the reasons for this discrepancy, rather than elucidating corrective measures.

Recently, to shorten the emergency laboratory test report time, point-of-care testing (POCT) has been introduced in some laboratories to replace conventional laboratory test methods, which has reduced test quality. In this study, more conventional and advanced instruments were adopted during IT-based optimisation, thereby greatly reducing the emergency laboratory test report time. Meanwhile, the emergency laboratory test report error rate also fell from 0.77‱ to 0.15‱ after IT-based optimisation, which eliminated emergency inspection report errors caused by the transmission of abnormal results from the equipment to the LIS. Nevertheless, after IT-based optimisation, certain defects were not eliminated and there are still some items that need to be manually entered. As a result, errors still occur occasionally due to other influencing factors before specimen analysis. In the future, an automatic review will be performed by the HIS system and if the laboratory test results are inconsistent with the comprehensive performance of the patients, inspectors will be prompted to recheck the results, which may reduce errors. Additionally, the intrinsic property (RBC ≈ Hb×3.5) will be examined to prevent the occurrence of events such as RBC reduction caused by condensation. Thus, we maintain that the extensive application of IT in laboratory testing will significantly improve test speed and quality.

IT-based optimisation shortens the emergency laboratory test report time and improves emergency laboratory test quality. Besides, the satisfaction levels of employees and patients also increase after the introduction of IT-based optimisation. In this study, employee satisfaction increased from 80.65 to 93.55% after optimisation, but the difference was not statistically significant. This may be due to false positives caused by the small number of employees. Specifically, the number of very satisfied employees increased by 29.03% (from 54.84 to 83.87%) after IT-based optimisation. Although the number of participants was relatively small, the difference was still statistically significant, and this result confirms that employee satisfaction rose as a result of IT-based optimisation. Additionally, several manual operations were replaced by automatic processing after IT-based optimisation. Hence, human error was avoided and employees could pay more attention to resolving complex problems. Also, even though the total workload increases, work intensity may decrease due to workflow optimisation and the implementation of informationisation and automation, which may be another reason for the improvement in employee satisfaction. Based on the integration of artificial intelligence and emergency laboratory tests, emergency laboratory test quality was comprehensively enhanced. Thus, employees obtained a higher sense of achievement, which may have contributed to the upturn in employee satisfaction. After IT-based optimisation, the overall satisfaction and high satisfaction of patients were greater than before IT-based optimisation. Besides, the absolute number and relative proportion of patients who were satisfied, generally satisfied, or dissatisfied also decreased significantly after IT-based optimisation. Reduced emergency laboratory test report times implied shorter waiting times, which may have relieved the anxiety of patients. Additionally, improved emergency test quality contributed to higher faith in the laboratory test results, which may have enhanced patient recognition of the test results. Therefore, the emergency laboratory test report time, test quality, and employee and patient satisfaction are all dependent on the overall quality of the emergency laboratory tests, which can be further improved through comprehensive optimisation.

Owing to constraints in resources, this study was configured as a single-center investigation, and the randomized controlled trial approach was not employed. Consequently, there exists the potential for some bias in the outcomes of this study, specifically in relation to the enhancement of emergency laboratory test report quality and the reduction of emergency laboratory test report time. Hence, a multi-center study is needed for further verification. Within this study, the IT-driven optimisation of emergency laboratory tests resulted in improvements not only in the quality of emergency laboratory test reports but also in the reduction of turnaround times for these reports. The production of precise and swift examination reports played a pivotal role in ensuring that emergency patients received effective care within the optimal rescue window. Moreover, it contributed to a reduction in the waiting period for patients and their families at the hospital. This contributed to alleviating their anxiety, thereby leading to a comprehensive improvement in their medical experience and satisfaction. Furthermore, the decreased work intensity and error rate among medical professionals engaged in emergency laboratory tests can elevate their sense of professional pride. As a result, the development of advanced emergency laboratory test capabilities is highly commended by clinicians, patients, and laboratory technicians.

## Conclusion

With the recent rapid advances in IT, the integration of IT into laboratory testing has become a popular research direction. The automation and standardisation of most stages in emergency laboratory testing can be realised by IT, which reduces the workload of employees and improves emergency laboratory test quality. However, the application of advanced technology in emergency laboratory testing requires the cooperation of IT engineers and inspectors. Thus, it is necessary to understand the cutting-edge knowledge related to information technology and laboratory testing to combine advanced theory with practical functions. Furthermore, training projects should also be implemented to promote the application of advanced technology, thereby maximising the advantages of IT. On that basis, the emergency laboratory test report time can be shortened, emergency laboratory test quality can be enhanced, and employee and patient satisfaction can also be improved. Moreover, the IT-based optimisation of the emergency test process should be conducted according to thorough evaluations of defects in existing laboratory processes. Meanwhile, it should be noted that IT-based optimisation is also a cumulative process. After the occurrence of any problems, it is necessary to conduct a comprehensive analysis to identify feasible solutions that contribute to optimisation.

## Data Availability

We declared that materials described in the manuscript, including all relevant raw data, will be freely available to any scientist wishing to use them for non-commercial purposes, without breaching participant confidentiality.
